# Arthroscopic treatment of a medial meniscal cyst using a posterior trans-septal approach: a case report

**DOI:** 10.1186/1758-2555-2-25

**Published:** 2010-10-12

**Authors:** Tsuyoshi Ohishi, Eiji Torikai, Daisuke Suzuki, Tomohiro Banno, Yosuke Honda

**Affiliations:** 1 Department of Orthopaedic Surgery, Enshu Hospital, Hamamatsu, Shizuoka, Japan

## Abstract

Arthroscopic partial menisectomy followed by cyst decompression is currently recommended for treatment of a meniscal cyst. However, it is doubtful whether partial menisectomy should be performed on cysts communicating with the joint in cases without a meniscal tear on its surface since meniscal function will be sacrificed. In this report, a meniscal cyst arising from the posterior horn of the medial meniscus without meniscal tear on its surface was resected using an arthroscopic posterior trans-septal approach. A 59 year-old male presented to our hospital with popliteal pain when standing up after squatting down. Magnetic resonance imaging revealed a multilobulated meniscal cyst arising from the posterior horn of the medial meniscus extending to the posterior septum with a grade 2 meniscal tear by Mink's classification. The medial meniscus was intact on the surface on arthroscopic examination. The meniscal cyst and posterior septum were successfully resected using a posterior trans-septal approach without harming the meniscus. This is the first report on a meniscal cyst being resected using an arthroscopic posterior trans-septal approach with a 9-month follow-up period.

## Background

Recently, selective menisectomy followed by decompression of the cyst is commonly performed in arthroscopic treatment of a meniscal cyst [1-3]. However, the surface of the meniscus is intact in the case of a grade 2 meniscal tear judged by magnetic resonance imaging (MRI) [4]. An operator might hesitate to perform partial resection of an intact meniscus in communication with a cyst since meniscal function could be sacrificed. In this paper, a meniscal cyst arising from the posterior horn of the medial meniscus was resected under arthroscopy, without menisectomy, using a posterior trans-septal approach. This is the first report on resection of a meniscal cyst using an arthroscopic posterior trans-septal approach.

## Case report

**Figure 1:**
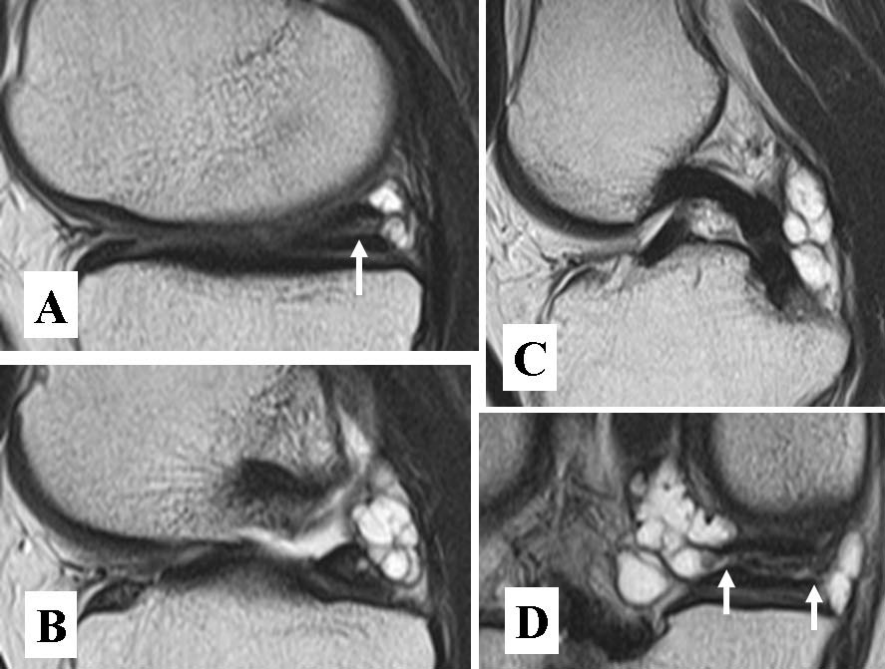
**Sagittal images (A,B,C) and coronal image (D) of T2 weighted MRI**. A horizontal meniscal tear is identified inside the meniscus in Figure 1-A (a white arrow). A multilobulated cyst is located just behind the medial meniscus and the PCL (B, C). The cyst is in communication with the meniscal tear on Figure 1-D (white arrows). Note that the horizontal meniscal tear has not extended to the tibial or femoral surface.

A 59-year-old jobless man presented at our hospital with a 2-month history of transient right popliteal pain when standing after squatting and while descending stairs. He was 170 cm tall and 55 kg in body weight and had not suffered from any antecedent trauma. Clinical examination of the right knee revealed a full range of motion without catching and locking episode. No swelling, warmness, erythema, tenderness (including the medial joint line) or hydrops was found around the knee. There was no anterior, posterior or lateral instability. He did not have pain during manual instability tests. McMurray's test reproduced pain but no click at the medial joint line. No mass was palpable around the knee. Standard radiographs showed no sign of osteoarthritis. An MRI revealed a grade 2 horizontal tear according to Mink's classification of the posterior segment of the medial meniscus [4] (Fig 1-A). A multilobulated meniscal cyst arising from the posterior horn of the medial meniscus to the posterior septum just behind the posterior cruciate ligament (PCL) was also detected (Fig 1-B, C). Communication tracts between horizontal tear of the meniscus and the cyst were identified on the MRI coronal plane (Fig 1-D).

**Figure 2:**
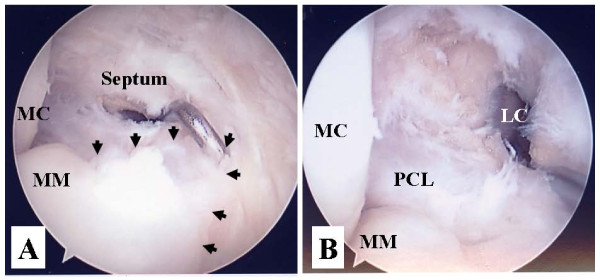
**Arthroscopic view from the posteromenial portal**. A probe is inserted via the trans-septal portal from the posterolateral portal (A). Proliferative synovial tissue (arrow heads) arising from the posterior horn of the medial meniscus (MM) expands to the septum. Resection of synovial tissue, cyst and posterior septum was completed (B). Posterior cruciate ligament (PCL) and lateral femoral condyle (LC) can be seen. MC: medial femoral condyle.

Arthroscopic surgery was performed under spinal anesthesia without a pneumotourniquet. The knee was flexed at more than 90 degrees on the operating table using a foot stopper. Anterior cruciate ligament, PCL and both lateral and medial meniscus were intact under arthroscopic examination. The posteromedial compartment was examined from the anterolateral portal through an intercondylar space. The surface of the posterior horn of the medial meniscus was intact and no cyst-like lesion was found on the edge of the posterior segment of the medial meniscus. Starting the procedure for making a trans-septal portal, the posteromedial and posterolateral portals were created according to the approach reported by Ahn et al [5]. After making a posterolateral portal, a rod with a sheath was inserted through the posterolateral portal to the septum. Pushing the sheath into the septum, the arthroscope was then inserted into the posteromedial portal. While maintaining the view of the medial side of a septum, a 1.5-3.0 mm Kirschner wire was pushed into the septum through the sheath from the posterolateral portal and then the septum was perforated. The Kirschner wire was pushed 2 or 3 times into the septum to enlarge the initial hole so that the switching rod could easily pass through the septum. Then, the switching rod was inserted from the posterolateral portal to the posteromedial portal via the trans-septal portal. Once the trans-septal portal was created, the arthroscope and instruments are easily interchangeable through the two posterior portals according to the posterior "back and forth" approach presented by Louisia et al [6]. Viewing from the posteromedial portal, proliferated synovial tissues expanding from the posterior horn of the medial meniscus to the posterior septum were found (Fig 2-A). The cyst and posterior septum were resected by a punch and a motorized shaver inserted from the posterolateral portal while viewing from the posteromedial portal. When the cyst ruptured, a clear mutinous fluid seeped from the cyst cavity. A meniscal tear was not found although the posterior edge of the medial meniscus was carefully examined by a probe after cyst resection (Fig 2-B).

**Figure 3:**
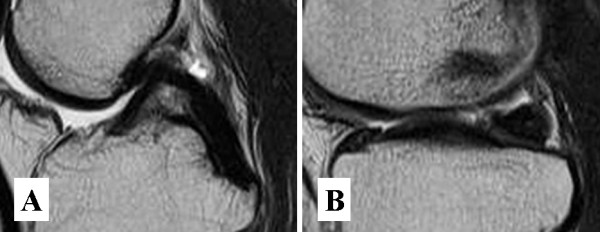
**Sagittal image of T2 weighted MRI examined 9 months after the operation**. The meniscal cyst has completely disappeared (A). Note that grade 2 signal intensity inside the meniscus decreased compared with abnormal signal intensity shown in Fig 1-A (B).

An MRI examination 9 months after the operation revealed that the meniscal cyst had disappeared and abnormal intensity inside the medial meniscus decreased (Fig 3-A,B). The patient's popliteal pain also disappeared completely during the 9-month follow-up period.

## Discussion

To our knowledge, this is the first report of a meniscal cyst being resected without harming the meniscus using a posterior trans-septal portal under arthroscopy.

Resection of a meniscal cyst involving the septum is difficult by the intercondylar approach from two anterior portals even with a posteromedial portal in addition for managing the procedure. Although the use of a 70-degree arthroscope can help the visualization of the posterior compartment, the whole area of the septum cannot be visualized since the camera head of an arthroscope is too near the septum from an anterior intercondylar approach. Although the septum can be viewed clearly from the posteromedial portal, it is cumbersome to manage the instruments to excise the cyst from anterior portals or from an additional high posteromedial portal. In our case, a posterior trans-septal approach was employed for the resection of the meniscal cyst as this approach allows an operator to identify the posterior edge of the medial meniscus, the septum and PCL and to treat the lesions involving those structures easily. A trans-septal approach was initially reported by Kim et al. in 1996 [7]. This technique is quite useful for accessing the posterior compartment of the knee, however the operator should bear in mind the risk of popliteal neurovascular injury when making a trans-septal portal. We have modified previously reported techniques to make a safe trans-septal portal. To avoid neurovascular complications, we always perforate the septum with a sheathed Kirschner wire in the lateral to medial direction while monitoring the tip of the Kirschner wire from the posteromedial portal since the popliteal neurovascular bundle is placed just posterior and lateral to the septum. We have used this approach for access to the posterior lesion in 95 knee cases and there have been no neurovascular complications from this procedure.

To date, articles on arthroscopic posterior trans-septal approaches for PCL reconstruction, suture of posterior horn of the medial meniscus or posterior capsules and excision of a ganglion cyst derived from the septum have been reported [8-12]. We have also used this posterior trans-septal approach to perform synovectomy of the posterior capsule, resection of the posterior horn of the medial meniscus, resection of PCL ganglion, and resection of free bodies in the posterior cavity. Although this technique allows us to manage lesions in the posterior cavity easily, it can be applied only when the lesion in the posterior cavity is difficult to access from the anterior intercondylar approach even with an additional posteromedial portal. An operator should always bear in mind that a neurovascular bundle is just behind the septum.

For the treatment of a meniscal cyst, previous reports recommended either a partial menisectomy with cyst decompression under arthroscopy or open cystectomy with a repair of the meniscus capsular attachment [3]. Almost all meniscal cysts are accompanied by some meniscal tear, including grade 2 lesions judged by MRI, although it is not obvious on the surface of the meniscus. In such cases as ours, an open cystectomy is recommended according to the therapeutic options for meniscal cysts [3]. However, substantial damages in the surrounding soft tissue, especially damage to the posterior capsule, at the popliteal fossa could be unavoidable by an open procedure since the lesion is deep under the skin and is not palpable on the skin. Recently, all arthroscopic procedures for selective menisectomy with cyst decompression or cyst drainage with suture of the torn meniscus were reported to preserve or restore a meniscal function [1,13,14]. Howe et al. demonstrated the excellent results for a long follow-up period by creating a small channel at the capsule adjacent to the cyst for decompression of the cyst into the joint without harming of the healthy meniscus [15]. The principle of our method in this case is almost same as Howe's one in terms of equalizing pressure between the meniscus and joint cavity by excising the cyst so as not to allow joint fluid re-enter to the torn meniscus through the communication tract.

Since MRI has become routinely available, more medial meniscal cysts, including asymptomatic ones, can be detected by MRI [16]. According to Campbell et al, 72 out of 109 meniscal cysts (66%) were located in the medial compartment and 53 of these medial meniscal cysts (74%) arose from the posterior horn of the medial meniscus [17]. Such evidence would indicate that meniscal cysts like our case are not rare and will increasingly be found. Popliteal pain in our patient might not be caused by a grade 2 meniscal tear but by impingement of the meniscal cyst between the posterior femoral condyle and posterior edge of the tibia since the patient felt pain after squatting down. A symptomatic meniscal cyst with an asymptomatic grade 2 meniscal tear should be treated by arthroscopic procedure without harming the meniscus.

Recurrence of meniscal cysts has been reported although long-term results of selective partial menisectomy with cyst decompression were satisfactory in previous papers [1,18]. Hulet et al. reported 11 out of 105 lateral meniscal cysts recurred after arthroscopic partial menisectomy followed by cyst decompression with an average 5-year follow-up [18]. They concluded that the reason for recurrence was mainly attributed to inadequate menisectomy performed so as not to damage the healthy part of the meniscus. Very few papers reported on the long-term results of meniscal cysts that were excised without managing the torn meniscus under arthroscopy [15]. Our case needs to be followed for a long period since menisectomy was not performed at all so as to completely preserve meniscal function.

## Conclusions

A posterior trans-septal approach is useful for resection of a meniscal cyst arising from the posterior horn of the medial meniscus, especially in the case where the lesion involves the posterior septum.

## Consent

Written informed consent was obtained from the patient for publication of this case report and any accompanying image. A copy of the written consent is available for review by the Editor-in-Chief of this journal.

## Competing interests

The authors declare that they have no competing interests.

## Authors' contributions

TO and ET carried out the surgical treatment and DS, TB and YH discussed the results and commented on the manuscript. All authors have read and approved the final manuscript.
